# Pharmacokinetic Correlates of the Effects of a Heroin Vaccine on Heroin Self-Administration in Rats

**DOI:** 10.1371/journal.pone.0115696

**Published:** 2014-12-23

**Authors:** Michael D. Raleigh, Paul R. Pentel, Mark G. LeSage

**Affiliations:** 1 Minneapolis Medical Research Foundation, Minneapolis, Minnesota, United States of America; 2 Hennepin Healthcare System, Minneapolis, Minnesota, United States of America; 3 Department of Medicine, University of Minnesota Medical School, Minneapolis, Minnesota, United States of America; 4 Department of Pharmacology, University of Minnesota Medical School, Minneapolis, Minnesota, United States of America; 5 Department of Psychology, University of Minnesota, Minneapolis, Minnesota, United States of America; Peking University, China

## Abstract

The purpose of this study was to evaluate the effects of a morphine-conjugate vaccine (M-KLH) on the acquisition, maintenance, and reinstatement of heroin self-administration (HSA) in rats, and on heroin and metabolite distribution during heroin administration that approximated the self-administered dosing rate. Vaccination with M-KLH blocked heroin-primed reinstatement of heroin responding. Vaccination also decreased HSA at low heroin unit doses but produced a compensatory increase in heroin self-administration at high unit doses. Vaccination shifted the heroin dose-response curve to the right, indicating reduced heroin potency, and behavioral economic demand curve analysis further confirmed this effect. In a separate experiment heroin was administered at rates simulating heroin exposure during HSA. Heroin and its active metabolites, 6-acetylmorphine (6-AM) and morphine, were retained in plasma and metabolite concentrations were reduced in brain in vaccinated rats compared to controls. Reductions in 6-AM concentrations in brain after vaccination were consistent with the changes in HSA rates accompanying vaccination. These data provide evidence that 6-AM is the principal mediator of heroin reinforcement, and the principal target of the M-KLH vaccine, in this model. While heroin vaccines may have potential as therapies for heroin addiction, high antibody to drug ratios appear to be important for obtaining maximal efficacy.

## Introduction

Heroin is the most widely abused illicit opioid worldwide [1] and its use has doubled over the last 10 years in the United States [2]. Pharmacotherapies available for the treatment of heroin addiction act at opioid receptors in the brain as either agonists to reduce cravings and prevent withdrawal (e.g. methadone), as antagonists to block heroin-reinforcing effects (e.g. naltrexone), or mixed agonist/antagonist (e.g. buprenorphine). These medications are effective but have side effects or constraints on their use that limit their appeal. Less than 20% of the patients in the US who might benefit from these medications are currently receiving them [2]. Additional treatment options are needed to address these problems.

Vaccination against heroin has been studied in animals as a mechanistically distinct treatment option for heroin addiction. Vaccination targets the drug itself rather than opioid receptors or other CNS targets. A variety of vaccines have been studied consisting of heroin or morphine conjugated to a foreign carrier protein. Antibodies elicited by vaccination bind heroin and its active metabolites [3]–[8] and reduce metabolite distribution to brain [9], [10]. Vaccines targeting heroin have shown preclinical efficacy for blocking a variety of heroin-induced behavioral effects, including heroin self-administration (HSA), anti-nociception, and locomotor activity in animals [3], [4], [6], [8], [11], [12].

The mechanism underlying the blockade of these behavioral effects is not completely understood for several reasons. First, heroin doses often exceed the binding capacity of available antibodies. Second, heroin is rapidly degraded and sequentially metabolized to its active metabolites 6-monoacetylmorphine (6-AM), morphine, and morphine-6-glucuronide. The metabolite 6-AM is considered important in mediating heroin’s early effects because it is found at higher levels in the brain than heroin or other metabolites shortly after heroin administration and has a higher affinity for the µ opioid receptor than heroin [13]–[15]. Immunization with the heroin vaccine M-KLH (morphine conjugated to keyhole limpet hemocyanin) was recently shown to bind heroin and its active metabolites in blood, reduce heroin-induced locomotor activity and anti-nociception, and to reduce the early distribution of heroin metabolites, but not heroin, to brain [10]. The effects of M-KLH on heroin and metabolite distribution in behavioral paradigms that involve repeated heroin doses and more closely model addiction, such as heroin self-administration, have not been studied. It is unclear whether vaccine-generated antibodies must bind heroin, its metabolites 6-AM and morphine, or all three to reduce heroin’s reinforcing effects. A better understanding of this interaction could help improve design of heroin vaccines. For example, if binding 6-AM in plasma is critical for reducing heroin’s reinforcing effects, vaccines that specifically target this heroin metabolite might be more effective than those designed for broader specificity.

To this end, the current study evaluated the effects of vaccination with the previously characterized M-KLH immunogen on the acquisition of HSA, maintenance of HSA during a dose-reduction protocol, and heroin-primed reinstatement of HSA. The pharmacokinetic correlates of vaccine effects on HSA were then studied by administering repeated i.v. heroin injections to vaccinated and control rats at doses comparable to those consumed during the HSA procedures. Results showed that vaccination with M-KLH altered HSA and reinstatement and that the magnitude of reduction in distribution of 6-AM to brain was consistent with and could account for the self-administration data.

## Materials and Methods

### 2.1. Ethics statement

This study was carried out in strict accordance with the recommendations in the Guide for the Care and Use of Laboratory Animals of the National Institutes of Health. Animal protocols were approved by the Minneapolis Medical Research Foundation Animal Care and Use Committee (protocol #08-10). All surgery was performed under droperidol/fentanyl anesthesia, animals were euthanized by CO_2_ inhalation using AAALAC approved chambers, and all efforts were made to minimize suffering.

### 2.2. Drugs and vaccine

Drugs were obtained through the NIH National Institute on Drug Abuse Drug Supply Program (Bethesda, MD) or Sigma-Aldrich (St. Louis, MO). Drug doses and concentrations are expressed as the weight of the base. A well-characterized heroin vaccine (M-KLH) was used, consisting of a morphine hapten conjugated via a tetraglycine linker to keyhole limpet hemocyanin (KLH) [9], [10]. M-KLH generates antibodies that have specificity towards heroin, 6-AM, morphine, and morphine-6-glucuronide but lacks specificity towards methadone, buprenorphine, naltrexone, oxycodone, and the endogenous opioid encephalin [10]. In this report, antibody specificity will be referred to as ‘morphine-specific’ to avoid confusion although these antibodies also bind the previously mentioned opioids. The morphine hapten was conjugated to bovine serum albumin (BSA) for use as a coating antigen for enzyme-linked immunosorbent assay (ELISA) and to KLH for immunization of rats [9], [16].

### 2.3. Animals and vaccinations

Male Holtzman rats (Harlan Laboratories, Madison, WI) weighing 350 g at arrival were single housed under a 12/12-hour standard light/dark cycle and were placed on restricted feeding (18 g/day). Testing occurred during the light phase. Rats received 25 µg of either M-KLH or unconjugated KLH on days 0, 21, and 42 for the drug distribution and HSA studies as well as once every three weeks throughout HSA testing. Immunogens were injected in a volume of 0.4 ml i.p. with Freund’s complete adjuvant (EMD Millipore, Billerica, MA) for the first immunization and Freund’s incomplete adjuvant (Sigma-Aldrich) for subsequent vaccine boosts.

### 2.4. Apparatus

Experimental sessions occurred in standard operant-conditioning chambers (ENV-007, Med Associates Inc., St. Albans, VT). The front panel contained two response levers and a stimulus light over each response lever. Each chamber was enclosed in a sound-attenuating box equipped with an exhaust fan that provided masking noise. Heroin was administered via an infusion pump (Model PHM-100-15, Med Associates Inc.). Presses on the left (active) lever produced an infusion of heroin while presses on the right lever were recorded but had no programmed consequence. A computer with MED-PC IV software (MED Associates, Inc.) was used for operating the apparatus and recording data.

### 2.5. Surgery

For HSA each rat was implanted with a chronic indwelling jugular catheter under intramuscular (i.m.) droperidol (1.25 mg/kg) and fentanyl (0.025 mg/kg) anesthesia. A silicon catheter (0.51 mm I.D.×0.94 mm O.D.) was inserted into the right jugular vein and advanced to the junction of the vena cava and the right atrium and sutured to tissue surrounding the vein. The catheter was tunneled subcutaneously (s.c.) to the back where it exited between the scapulae and attached to a guide cannula mounted in a harness assembly (VAH95AB, Instech Laboratories Inc., Plymouth Meeting, PA) on the back of the rat. A stainless steel spring tether attached to the guide cannula allowed connection to a fluid swivel for heroin administration. Rats were allowed to recover for approximately 1 week after surgery, during which each rat received an antibiotic (20 mg/kg enrofloxin, s.c.) immediately followed by intravenous (i.v.) infusions of heparinized saline (30 units/ml) for three days to maintain catheter patency. Heparinized saline infusions were continued after each session for the remainder of the experiment.

### 2.6. Antibody characterization

ELISA plates were coated with 5 ng/well morphine conjugated to BSA via a tetraglycine linker in carbonate buffer at 9.6 pH and blocked with 1% gelatin. Goat anti-rat antibodies conjugated to horseradish peroxidase were used as secondary antibodies. Morphine-specific antibody concentrations were calculated based on a previously standardized monoclonal antibody assay [9].

### 2.7. Stoichiometric relationships

The total number of moles per kilogram of morphine-specific IgG in rats vaccinated with M-KLH was estimated as the product of the reported IgG volume of distribution (131 ml/kg) in rats and the plasma antibody concentration, assuming a molecular weight of 150 kDa for IgG. The number of IgG binding sites was calculated as twice that number [9], [17]. The numbers used for calculating molar binding sites in Table 1 were the mean IgG binding sites in each group.

**Table 1:** Molar ratios of calculated opioid-specific IgG binding sites to the administered heroin dose.

Experiment	Total heroin dose (µmol/kg)	Total opioid-specific IgG binding sites (µmol/kg)	Molar Ratio of Drug Dose: IgG Binding Sites
Behavioral studies			
HSA acquisition^1^			
KLH (1.0 mg/kg/2-hr)	2.7	–	–
M-KLH (1.9 mg/kg/2-hr)	5.1	0.82	6.2
HSA dose-reduction			
0.003 mg/kg/inf (16 inf)	0.1	0.82	0.1
0.01 mg/kg/inf (22 inf)	0.6		0.7
0.03 mg/kg/inf (54 inf)	4.4		5.4
0.06 mg/kg/inf (35 inf)	5.7		7.0
Reinstatement	1.6^2^	0.82	1.7
**Drug distribution studies**			
0.5 mg/kg/1-hr	1.4	0.72	1.9
1.0 mg/kg/1-hr	2.7	0.63	4.3

IgG binding site concentrations are the means for all vaccinated rats in that experiment. Doses listed for the HSA studies are the mean total heroin intake over the 2-hr self-administration session. Doses listed for the drug distribution studies are the total amount of heroin administered over the 1-hr experiment.

1 Last three HSA sessions at 0.06 mg/kg/inf heroin during FR3.

2 s.c. administration, all other heroin doses administered i.v.

### 2.8. Drug level analysis

Plasma and brain heroin, 6-AM, and morphine concentrations (bound + unbound) were measured by liquid chromatography/mass spectrometry by a validated method described previously [18]. Limit of quantitation was 5 ng/ml for heroin and 6-AM and 10 ng/ml for morphine. Samples were analyzed within 3 days of extraction under conditions that minimized their degradation [18]. Morphine-6-glucuronide was not measured because it is not appreciably formed in rats [19]. Briefly, trunk blood was collected in a syringe containing 4 mg/ml of ice-cold NaF and heparin (100 IU/ml) and centrifuged immediately at 3100×*g* for 3 minutes at 4°C. Plasma was diluted 1∶1 with ice-cold 10 mM formate buffer (pH 3.0) prior to extraction. Brains were rinsed with 10 mM formate buffer (pH 3.0), and four parts (by weight) of 10 mM formate buffer pH 3.0 was added to each sample. Samples were homogenized (Model PT 10–35 with PCU 11, Brinkmann Instruments Inc., Riverview, Fl) for 30–40 seconds and stored for 5–60 min at −20°C until extraction.

### 2.9. Heroin self-administration protocol

Prior to the acquisition of HSA, rats were vaccinated with M-KLH (n = 13) or KLH (control, n = 12). Three days after the third vaccination, jugular catheters were implanted. Blood was drawn from the indwelling cannulas one week following the third vaccination and also one week following the final vaccination (a total of 9 vaccinations) for antibody characterization.

#### 2.9.1. Acquisition of HAS

Approximately 1 week after surgery, rats were given 2 hr/day access to response-contingent heroin infusions (0.06 mg/kg/infusion) delivered at 50 µl/kg per second [20]. This unit dose was chosen because it has been shown to maintain HSA in rats [20]. Infusions were available under a fixed-ratio (FR 1) schedule for 11 days, then FR 2 for 5 days, and then FR 3 for 5 days. The active (left) lever was baited with food powder on the first day at FR 1 to facilitate contact with the lever and the reinforcement contingency. Data from this session was not included in the analysis. Sessions began with onset of the stimulus light above the active response lever. When the response requirement for drug was met on the active lever, the stimulus light was extinguished during the infusion and a subsequent 5-s timeout during which responses had no programmed consequence. Following the timeout, the stimulus light was illuminated indicating availability of the next infusion. The criteria for acquisition were a minimum of 10 heroin infusions/session and a ratio of active to inactive lever presses of at least 2∶1 for the last three consecutive sessions at FR 3. These criteria are based on pilot data indicating that discriminated lever pressing was not typically observed when fewer infusions were earned per session. Catheter patency was checked weekly (Fridays) and at the end of the experiment by observing rapid anesthesia (within 3–5 sec) upon i.v. infusion of methohexital (1.5 mg). Rats that failed this patency check (n = 8) or had other problems (e.g. pulled out the catheter (n = 1) or exhibited significant self-mutilation (n = 2)) at any point in the acquisition protocol were excluded from the experiment, leaving a final sample size of n = 6 in the KLH group and n = 8 in the M-KLH group for this phase of the study.

#### 2.9.2. Dose-reduction and reinstatement of HAS

Following the last FR 3 session, the heroin unit dose was decreased every five sessions to 0.03, 0.01, 0.003, and 0 mg/kg/infusion to obtain a heroin dose-response curve in rats that acquired HSA. After active-lever responding stabilized under extinction conditions (less than 30% of baseline and no significant trend for three consecutive sessions), each rat received a priming injection of saline or heroin (0.6 mg/kg, s.c.) 15 min before an extinction session to examine vaccine effects on reinstatement of HSA responding. Reinstatement tests were conducted on Tuesdays and Fridays provided that responding was stable during the previous session, and the order of the priming dose was counterbalanced across subjects. Each dose was evaluated once. Data for the lowest unit dose were excluded for two KLH control rats because one rat’s catheter lost patency at this dose and the other developed an ear infection. Data for the lowest unit dose were also excluded for one M-KLH rat that pulled out its catheter. As a result, the final sample size at the 0.003 mg/kg unit dose was n = 4 KLH rats and n = 7 M-KLH rats. One M-KLH rat died during the methohexital infusion while checking catheter patency before reinstatement testing could be completed, leaving a final sample size of n = 6 M-KLH rats for this phase.

### 2.10. Measurement of heroin and metabolite distribution

Separate groups of rats were used to study effects of vaccination on heroin and metabolite distribution under conditions approximating those observed during HSA as described above. Two groups of 6 rats vaccinated with M-KLH and two control groups vaccinated with KLH received 8 equally spaced i.v. infusions of either 0.17 µmol/kg (0.0625 mg/kg/inf or 0.5 mg/kg/hr) or 0.34 µmol/kg (0.125 mg/kg/inf or 1 mg/kg/hr). These doses were chosen because they approximated the mean heroin exposure during HSA of 0.5 mg/kg/hr heroin in control rats and 1.0 mg/kg/hr heroin in vaccinated rats (during FR3 responding for heroin at 0.06 mg/kg/inf). To avoid respiratory depression 1-hr drug distribution sessions were performed at approximately half of the total doses administered in the 2-hr HSA sessions. This was because animals in the drug distribution studies were drug naïve and anesthetized throughout the entire procedure. Animals were anesthetized 1 week after their final vaccination and blood was withdrawn from the jugular catheters for antibody characterization prior to drug administration. Four minutes after the first infusion of heroin, blood was drawn via the tail vein to quantitate heroin, 6-AM, and morphine levels after a single heroin dose. Rats were decapitated for collection of trunk blood and brain four minutes after infusion of the 8^th^ heroin dose to measure drug levels after all doses had been delivered.

### 2.11. Statistical analysis

The effect of treatment on HSA was analyzed using two-way analysis of variance with group as a between-subjects factor and day as a within-subject factor followed by multiple t-tests between groups at each session using the False Discovery Rate (FDR) approach with the FDR Q = 5%, a recommended approach when large numbers of post-hoc comparisons are of interest [21]. When group variances were unequal, t-tests were performed using Welsh’s correction. Because data from the dose reduction phase were not normally distributed, medians are reported instead of means and comparisons between groups at each unit dose were made via nonparametric Mann-Whitney U tests. In addition, a Chi-Square test was used to compare the proportion of rats exhibiting a reduction in infusion rate at the two lowest unit doses versus their baseline infusion rate at the 0.06 mg/kg dose. To examine the effects of heroin priming during extinction, the mean number of active lever presses following saline and heroin priming injections in KLH and M-KLH rats was analyzed using two-way repeated-measures ANOVA followed by Bonferroni-corrected t-tests.

To determine whether vaccination reduced the reinforcing efficacy of heroin independent of reducing heroin’s potency, exponential demand curve analysis was performed on heroin intake during HSA unit dose reduction as previously described [22], [23]. Briefly, an exponential demand equation, **log Q = log Q_0_+** ***k*** **(** ***e*** ^−α^*^Q^_0_*^C^−1), was used to describe the relationship between heroin consumption and unit price (FR/unit dose). The dependent variable, **Q**, is the quantity consumed. The independent variable, **C**, is the cost of heroin based on the unit price. The free parameters, **Q_0_** and **α**, are estimated from the best-fit function and refer to the maximum level of consumption at zero price (i.e., level or “intensity” of demand) and the rate of change in consumption with increases in unit price, respectively. The range of the exponential function, **k**, is a constant specifying the range of consumption in log units. The **k** value is held constant across data sets being compared (set to 1.2 in the present study) because changes in **k** impact the value of **α**. The value of **α** is inversely related to reinforcing strength so that drugs that produce rapidly declining (elastic) demand curves have higher **α** values and lower reinforcing strength than demand curves with slower declining (inelastic) demand curves. In the present context, vaccines that increase **α** values for heroin consumption would be those that increase sensitivity of HSA to price (increase elasticity of demand), suggesting that vaccination reduces the reinforcing efficacy or essential value of heroin, independent of changes in heroin potency [24]. Demand curve analysis was conducted using an exponential demand template for GraphPad Prism 6 software (GraphPad Software, Inc., La Jolla, CA) provided by Dr. Steven Hursh from the Institutes for Behavioral Resources (Baltimore, MD). Differences in **Q_0_** and **α** were compared between groups via a sum-of-squares F-test.

Effects of vaccination on drug distribution between M-KLH and KLH control groups were analyzed using unpaired t-tests. Differences in antibody titers and concentrations between groups were compared using one-way analysis of variance. Relationships between antibody levels and opioid distribution were analyzed using linear regression.

## Results

### 3.1. Serum antibody characterization

Vaccination with M-KLH elicited high morphine-specific antibody titers and concentrations in all groups and averaged 400±200×10^3^ and 420±170 µg/ml (mean ± SD), respectively. Titers in the M-KLH vaccinated rats during HSA were 460±260×10^3^ and the antibody concentrations were 470±220 µg/ml (mean ± SD). Titers in the M-KLH vaccinated rats in the drug distribution study were 350±150×10^3^ and antibody concentrations were 390±130 µg/ml (mean ± SD). Antibody titers, and concentrations calculated from these, were not statistically different (p = 0.27) between vaccinated rats in the HSA and drug distribution groups.

### 3.2. Vaccine effects on acquisition of HSA (heroin unit dose 0.06 mg/kg/inf)

**Figure 1:**
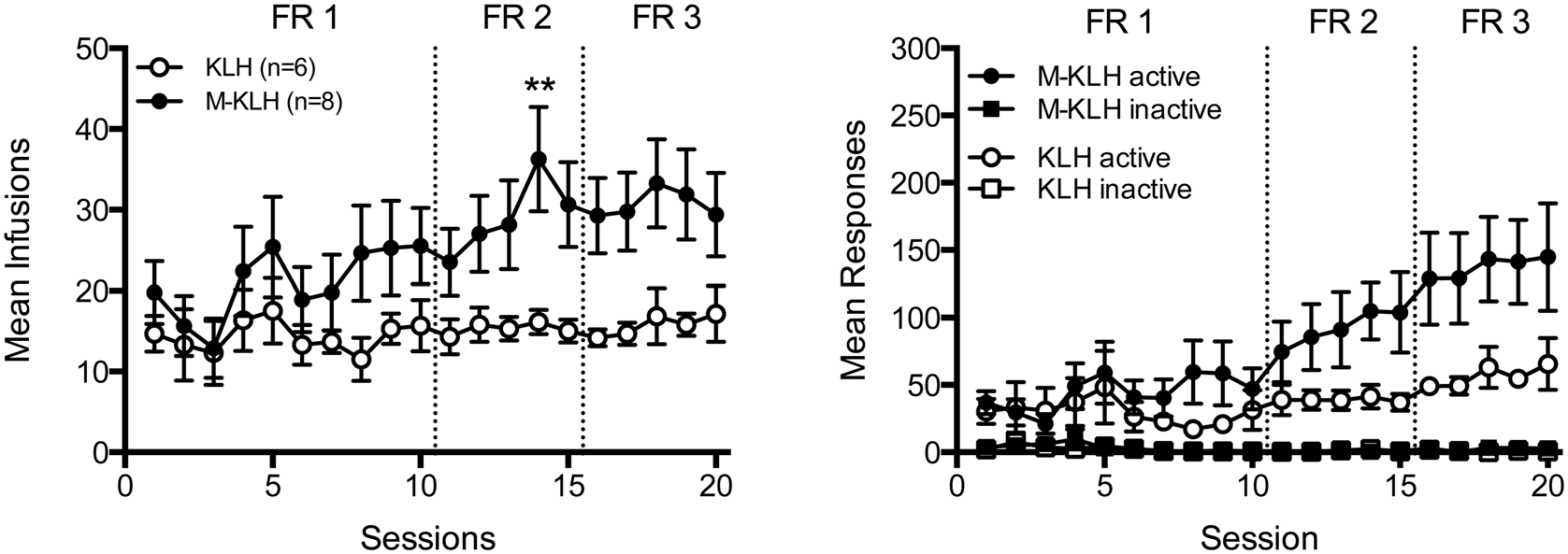
Effect of vaccination on acquisition of HSA. Mean (± SEM) infusions (0.06 mg/kg/inf heroin; left panel) and mean (± SEM) lever presses (right panel) on the active and inactive lever per session during the acquisition phase. Different from KLH, **p<0.01.

There was no significant main effect of vaccination on the mean number of infusions per session during the acquisition phase, but significant effects of session (F = 4.66, p<0.001) and vaccination×session interaction (F = 2.52, p<0.001) (Fig. 1, left panel). Vaccinated rats generally showed a higher mean number of infusions compared to controls, but there was a significant difference between groups only during one session at FR2 (t = 3.16, p<0.01). Analysis of mean active lever responses (Fig. 1, right panel) showed no significant effect of vaccination, but a significant effect of session (F = 9.61, p<0.001) and vaccination×session interaction (F = 3.45, p<0.001). There was no significant difference in the mean number of active lever presses between groups during any single session. Only one rat from the entire study, a vaccinated rat, failed to meet acquisition criteria for HSA. When this rat was removed to clarify the apparent vaccination-induced compensatory increase in HSA, there was a significant main effect of vaccination (F = 13.48, p<0.01), session (F = 5.51, p<0.001), and vaccination×session interaction (F = 3.19, p<0.001). Mean HSA infusions and active-lever response rates at the end of the acquisition phase (last three sessions at FR3, all rats included) were significantly higher in vaccinated rats compared to controls (t = 2.65, p<0.05 and t = 2.43, p<0.05, respectively). These measures were not significantly correlated with serum antibody concentrations. The mean total amount of heroin infused over 2-hr during the last three sessions at FR3 was 1.89 (±0.31 SEM) mg/kg in the vaccinated rats and 1.0 (±0.13 SEM) mg/kg in the control rats.

### 3.3. Vaccine effects during HSA dose-reduction

**Figure 2:**
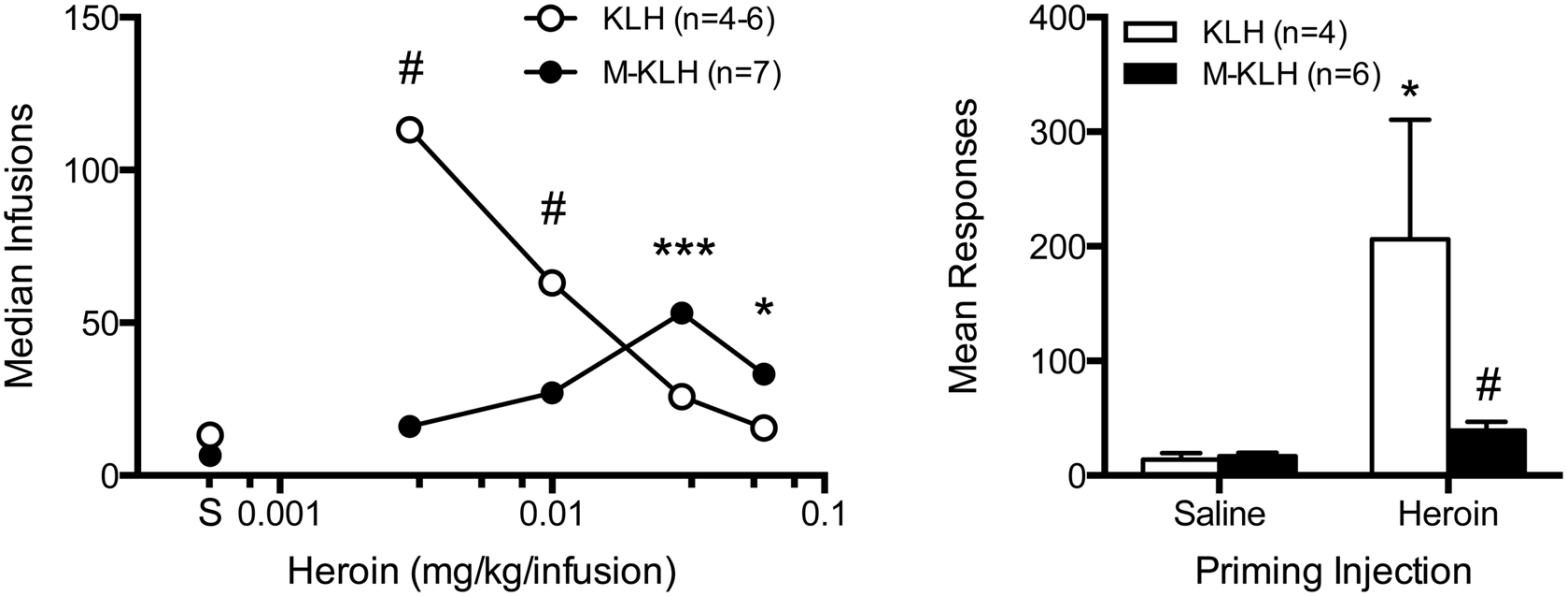
Effects of vaccination on HSA during dose reduction and reinstatement. Median total heroin infusions in rats that acquired HSA and completed the dose-reduction protocol (left panel). Rats were trained at a heroin unit dose of 0.06 mg/kg/inf. The heroin dose was sequentially decreased every 5 days to 0.03, 0.01, 0.003 and 0 mg/kg to obtain a dose-response curve. Median infusions compared to KLH *p<0.05, ***p<0.001. Proportion (*Χ^2^*) below 0.06 mg/kg compared to M-KLH #p<0.05. Blockade of reinstatement of heroin responding in vaccinated rats (right panel). Mean (± SEM) active lever presses during extinction in vaccinated and control rats after a s.c. priming injection of saline or 0.6 mg/kg heroin. *p<0.05 compared to saline control and #p<0.05 compared to KLH control.

Vaccinated rats showed higher median HSA rates at the 0.03 and 0.06 mg/kg unit doses compared to controls (U = 0.0, p<0.001; U = 7.5, p<0.05; respectively) (Fig. 2, left panel). Although vaccinated rats showed lower median rates at the 0.01 and 0.003 mg/kg unit doses, these differences were not statistically significant. Nonetheless, a greater proportion of vaccinated rats exhibited infusion rates below their 0.06 mg/kg baseline compared to controls at the 0.01 (4/8 vs 0/6, respectively, *X^2^* = 4.20, p<0.05) and 0.003 mg/kg unit doses (5/7 vs 0/4, respectively, *X^2^* = 5.24, p<0.05). During the reinstatement test (Fig. 2, right panel), there was a significant main effect of dose (F = 6.97, p<0.05), but the treatment and interaction effect only approached significance (p<0.1). Post-hoc comparisons showed the s.c. priming injection of heroin produced a significant increase in mean active lever responses in control rats, but not vaccinated rats (t = 3.06, p<0.05). Moreover, the mean number of active lever presses during the heroin priming test was significantly lower in M-KLH rats compared to controls (t = 2.84, p<0.05).

**Figure 3:**
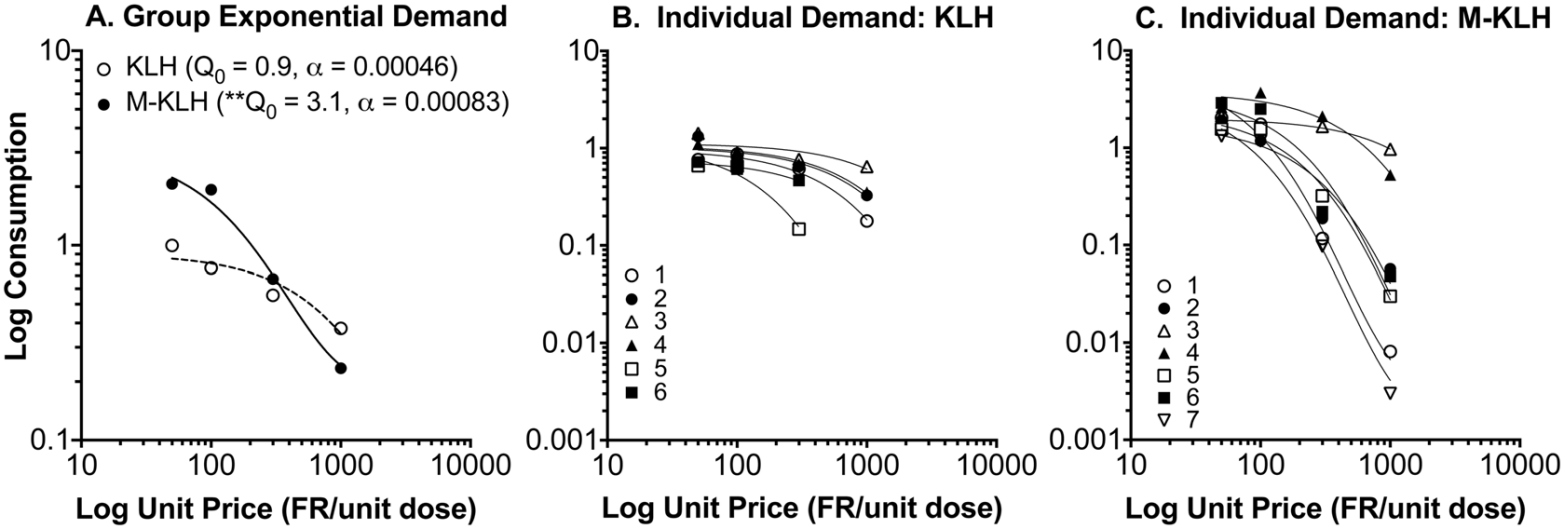
Consumption of heroin in vaccinated and control rats as a function of unit price (FR/unit dose). Panel A represents absolute log consumption for each group and panels B and C represent individual subject data. Vaccinated rats had a higher **Q_0_** (*p<0.01) compared to controls, but there was no difference in **α**.

Fig. 3 shows group and individual-subject demand curves describing the changes in heroin intake during the dose reduction phase. Decreases in heroin consumption with increases in unit price (i.e., decreases in unit dose) were well described by the exponential demand equation for both group and individual subject data (r^2^ = 0.88 (±0.06 SEM) and 0.95 (±0.02 SEM) for control and vaccinated rats, respectively). F-test analysis indicated a significantly higher **Q_0_** (initial level of demand) in vaccinated compared to control rats (**Q_0_** = 3.1 (±0.51 SEM) vs 0.91 (±0.11 SEM), respectively, F = 39.0, p<0.01), but no difference in **α** values (elasticity of demand) (**α** = 0.00083 vs 0.00046, respectively).

### 3.4. Effect of vaccination on heroin and metabolite distribution

All plasma heroin concentrations in control rats were below the assay limit of quantitation of 5 ng/ml, but could be estimated because they were above the limit of detection (S1 Table).

#### 3.4.1. Vaccine effects on opioid distribution in plasma after the 1st i.v. infusion of heroin

**Figure 4:**
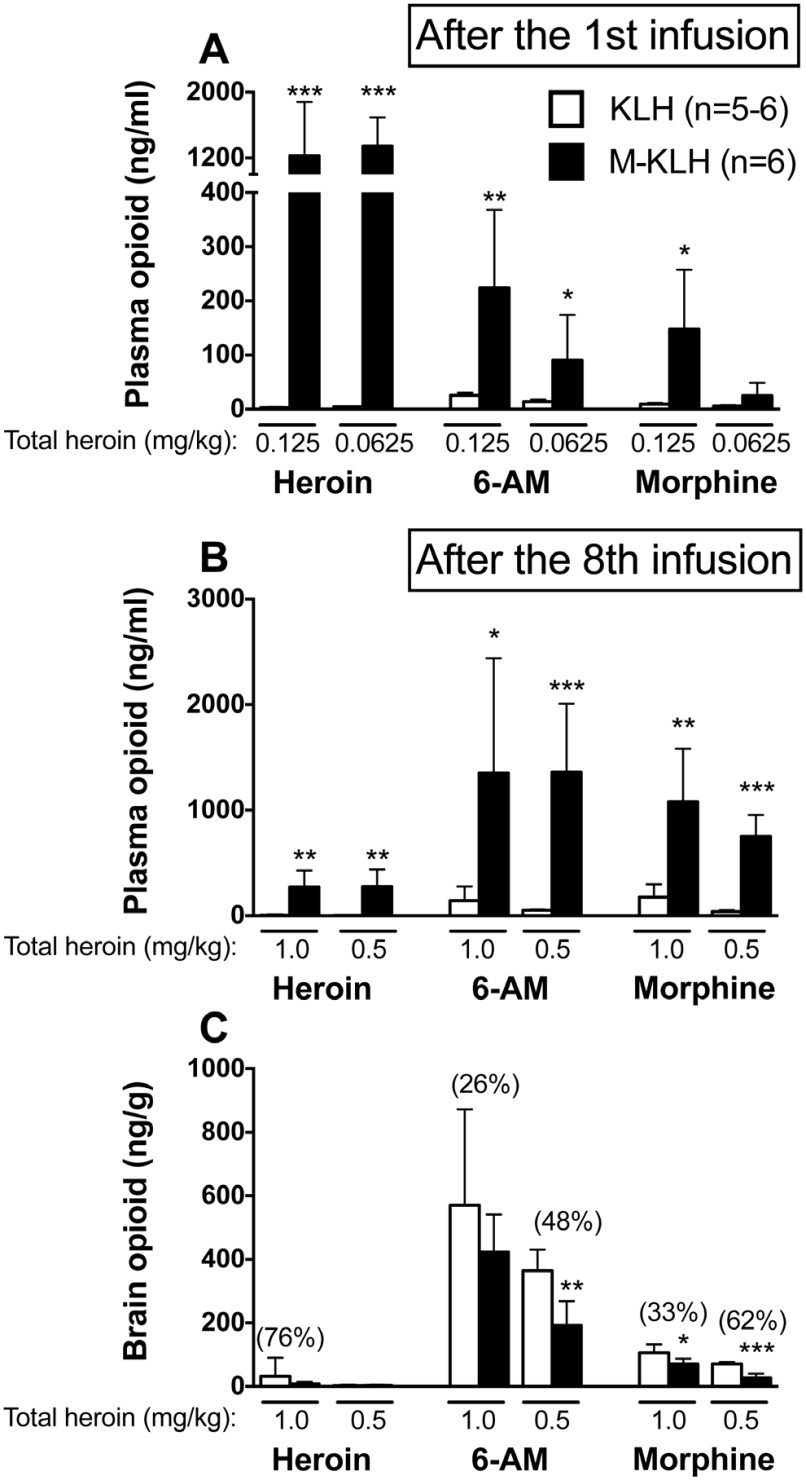
Distribution of heroin and its metabolites in plasma and brain after i.v. infusion of 0.125 or 0.0625 mg/kg heroin. Plasma (A) opioid concentrations (mean ± SD) after the 1^st^ infusion of heroin. Plasma (B) and brain (C) opioid concentrations (mean ± SD) after the 8^th^ infusion of 0.125 or 0.0625 mg/kg heroin (cumulative doses of 1.0 and 0.5 mg/kg heroin, respectively). Vaccination increased retention of heroin and its metabolites in plasma after the 1^st^ as well as after all 8 heroin infusions, at both doses. Vaccination also reduced opioid concentrations in brain after all 8 heroin infusions, though effects were greatest at the lower heroin unit dose. Numbers in parentheses are the percent decrease compared to controls. *p<0.05, **p<0.01, and ***p<0.001 compared to KLH controls. Some drug levels were quite low. See S1 Table for exact values.

*1^st^ infusion of 0.125 mg/kg heroin:* Mean plasma heroin concentrations were 410-fold greater in vaccinated rats compared to controls (p<0.001, Fig. 4A). Mean plasma 6-AM concentrations were 9-fold higher in vaccinated rats compared to controls (p<0.01). Plasma morphine concentrations were 16-fold higher in vaccinated rats compared to controls (p<0.05).

*1^st^ infusion of 0.0625 mg/kg heroin:* Mean plasma heroin concentrations were 335-fold greater in vaccinated rats compared to controls (p<0.001, Fig. 4A). Mean plasma 6-AM concentrations were 6-fold higher in vaccinated rats compared to controls (p<0.05). Plasma morphine concentrations were 5-fold higher in vaccinated rats compared to controls, but this difference was not significant.

#### 3.4.2. Vaccine effects on cumulative opioid distribution in plasma after the final (8th) i.v. infusion of heroin

*Final (8^th^) infusion of 0.125 mg/kg heroin (1.0 mg/kg cumulative dose):* Mean plasma heroin concentrations were 39-fold greater in vaccinated rats compared to controls (p<0.01, Fig. 4B). Mean plasma 6-AM concentrations were 9-fold higher in vaccinated rats compared to controls (p<0.05). Plasma morphine concentrations were 6-fold higher in vaccinated rats compared to controls (p<0.01).

*Final (8^th^) infusion of 0.0625 mg/kg heroin (0.5 mg/kg cumulative dose):* Mean plasma heroin concentrations were 93-fold greater in vaccinated rats compared to controls (p<0.01, Fig. 4B). Mean plasma 6-AM concentrations were 28-fold higher in vaccinated rats compared to controls (p<0.001). Plasma morphine concentrations were 19-fold higher in vaccinated rats compared to controls (p<0.001).

#### 3.4.3. Vaccine effects on cumulative opioid distribution in brain after the final (8th) i.v. infusion heroin

*Final (8^th^) infusion of 0.125 mg/kg heroin (1.0 mg/kg cumulative dose):* Mean brain heroin concentrations were not significantly reduced in vaccinated rats compared to controls (p = 0.4) but all levels were very low (Fig. 4C). Brain 6-AM concentrations were reduced by 26% in vaccinated rats compared to controls, but this effect was not significant. Brain morphine concentrations were reduced by 33% in vaccinated rats compared to controls (p<0.05).

*Final (8^th^) infusion of 0.0625 mg/kg heroin (0.5 mg/kg cumulative dose):* Brain heroin concentrations were not reduced in vaccinated rats compared to controls (p = 0.7) but all levels were very low (Fig. 4C). Brain 6-AM concentrations were reduced by 48% in vaccinated rats compared to controls (p<0.01). Brain morphine concentrations were reduced by 62% in vaccinated rats compared to controls (p<0.001).

**Figure 5:**
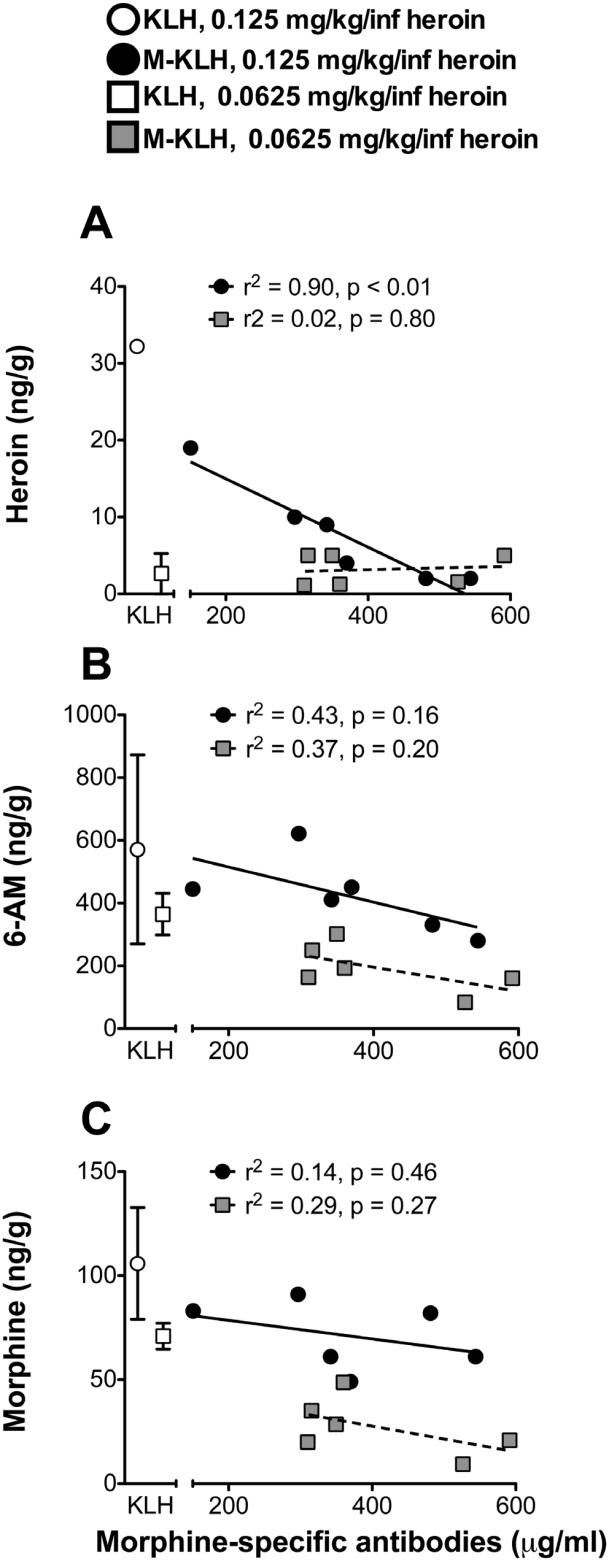
Relationship between morphine-specific antibody concentrations and opioid concentrations in brain. Heroin (A), 6-AM (B), and morphine (C) concentrations in brain after the final (8^th^) i.v. infusion of 0.125 or 0.0625 mg/kg heroin in vaccinated rats. KLH data shown as mean ± SD. Deviation not shown for ‘KLH, 0.125 mg/kg/inf heroin’ in the top panel because it was large (32±58 ng/g, mean ± SD).

Higher morphine-specific antibody concentrations were associated with lower opioid concentrations in brain, though a significant effect was seen only with brain heroin concentrations (r^2^ = 0.90, p<0.01) after the cumulative 1.0 mg/kg heroin dose (Fig. 5).

### 3.5. Stoichiometry

The total molar heroin dose administered during HSA acquisition, reinstatement, and during drug distribution studies exceeded the available morphine-specific IgG binding sites (Table 1). In contrast, the molar heroin dose administered in the dose-reduction protocol was lower than the available morphine-specific IgG binding sites. In general, higher molar ratios of antibody concentrations to drug doses were associated with lower brain opioid concentrations in the drug distribution studies as well as greater effects on HSA during dose-reduction.

## Discussion

The goal of this study was to investigate the effects of vaccination with M-KLH on HSA and to understand how these effects might be explained by associated changes in heroin and metabolite distribution. The main findings were 1) vaccination blocked heroin-primed reinstatement of HSA responding during extinction, 2) vaccination effects on HSA were dose-related, with a decrease in HSA at low heroin unit doses and a compensatory increase in HSA at high unit doses, and 3) changes in brain 6-AM concentrations after vaccination were consistent with the observed changes in HSA at the 0.06 mg/kg/inf heroin unit dose. Vaccination also substantially reduced morphine distribution to brain but morphine levels were lower than those of 6-AM and probably contributed less to the behavioral effects seen in this study. These findings support a potential role for vaccination in treating heroin abuse and provide evidence that 6-AM is a key mediator of the reinforcing effects of heroin.

Heroin-primed reinstatement of HSA was blocked in vaccinated rats at a substantial heroin dose (0.6 mg/kg, s.c.). This dose is within the range of rewarding doses in humans [25] and suggests that heroin vaccines could play a role in relapse prevention. This finding is consistent with those obtained using other heroin vaccines [6], [11].

The more complex, dose-related effects of vaccination on HSA are most readily explained by vaccination reducing the potency of heroin, as indicated by the rightward shift in the HSA dose-response curve. This effect can be attributed to the binding of antibodies to heroin and its active metabolites in serum, resulting in lower opioid concentrations in brain. Consistent with this interpretation, vaccinated rats receiving 0.06 mg/kg/inf heroin self-administered as much heroin as controls receiving 0.03 mg/kg/inf. Similarly, vaccinated rats receiving 0.03 mg/kg/inf heroin self-administered as much heroin as controls receiving 0.01 mg/kg/inf. Moreover, there was a significant difference in **Q_0_** (increased baseline level intake,) but not **α** (no change in sensitivity to price) in the demand curve analysis. Because **α** provides a standardized measure of reinforcing efficacy that controls for potency differences between groups, this analysis further confirms that vaccination reduced the potency of heroin rather than its reinforcing efficacy. In support of this, brain 6-AM concentrations in vaccinated rats receiving 1.0 mg/kg heroin were similar to those of control rats receiving 0.5 mg/kg. Although heroin and metabolite distribution to brain were not studied at lower heroin doses, vaccine-elicited antibodies presumably reduced brain 6-AM concentrations sufficiently to suppress HSA.

A previous study examining the effects of vaccination with M-KLH on the distribution of a single heroin dose in rats found a marked reduction of 6-AM distribution to brain. Morphine distribution to brain was also reduced but levels were substantially lower than those of 6-AM. Heroin distribution to brain was not reduced despite appreciable binding of heroin in serum [10] but the levels were quite low. The current study of multiple heroin doses is similar, in that 6-AM levels in brain predominated over those of heroin or morphine, but suggests that heroin distribution to brain was also reduced by vaccination. The mean reduction in brain heroin concentration was not significant but there was a significant inverse correlation between serum antibody concentrations and brain heroin concentrations. Because 6-AM levels in brain predominated, these findings support the primary role of 6-AM in mediating the behavioral effects of heroin, and as the most important drug target for the antibodies generated by vaccination with M-KLH.

It was recently shown that a monoclonal antibody with specificity for 6-AM over heroin or morphine reduced locomotor activity following a single heroin dose in mice [26]. This finding established antibody-mediated reduction in 6-AM brain concentrations as sufficient to alter acute (single dose) heroin-induced behavior. The current study extends and supports this observation by showing compatible effects on heroin and metabolite distribution in the setting of multiple doses, and at a dosing level sufficient to sustain heroin-reinforced behavior. It is possible that reducing morphine distribution to brain contributes to M-KLH efficacy for altering heroin-associated behaviors, but the lower total levels of morphine in brain compared to 6-AM are most consistent with morphine having a lesser role over the time periods that have been studied. It is unlikely that reduced heroin distribution to brain directly reduces heroin-associated behaviors because heroin levels were quite low and it is a weak mu opioid receptor agonist compared to 6-AM or morphine [27]. However, it is possible that binding of heroin by antibody in serum slows its conversion to 6-AM and contributes to the overall behavioral efficacy of M-KLH by this means. Morphine-6-glucuronide was not measured in this study because it is not produced in rats. However, morphine-specific antibodies generated by vaccination with M-KLH have been shown to bind morphine-6-glucuronide in vitro [10].

Vaccination remained effective even when the total amount of drug administered exceeded that of the available antibodies. However, vaccine effects were most prominent when heroin unit doses were lower than the binding capacity of the available antibodies. An important consideration is that some estimates of single doses used by addicts are as high as 2 mg/kg/inf (and up to 7 mg/kg/day) [25]. However, these estimates are from self-report, with the purity of heroin unknown. The rewarding dose of heroin in the setting of clinical laboratory studies is lower than these reports, in the range of 0.35–1.43 mg/kg [28], which is similar to some of the doses administered in this study.

These findings, along with the substantial reduction of HSA after M-KLH vaccination at the lower heroin unit doses and the blocking of reinstatement of heroin responding by M-KLH, support the ability of M-KLH to alter heroin-associated addiction-relevant behaviors in rats. They are largely consistent with the findings of others with several different heroin vaccines, which also demonstrated reductions in the reinstatement or re-acquisition of heroin self-administration, as well as attenuation of heroin-induced analgesia and locomotor activity [3], [4], [6], [11], [12]. The current study extends these reports by examining heroin self-administration over a range of heroin unit doses. While it adds support to the efficacy of these vaccines in animals, our findings also caution that high antibody levels may be necessary to achieve efficacy at high levels of heroin intake. It is difficult to quantitatively extrapolate this rat model of reinforcement to humans since there is no comparable human experience with a heroin vaccine. The amount of antibody required in humans to achieve a therapeutic benefit is ultimately an empirical question. The availability of vaccines such as M-KLH that have large effects on heroin pharmacokinetics provides a tool for studying this question.

It is difficult to directly compare effects of M-KLH with those of other vaccines because of immunological (e.g., vaccine doses, vaccination schedules, and hapten-conjugate structures) and behavioral (e.g. acquisition verses reacquisition, FR schedules, and session length and time-out) differences between studies. However, one common measure used to test vaccine efficacy in several studies was anti-nociception, where rats received 1 mg/kg heroin 30 minutes prior to placement on a 54°C hot-plate to measure latency to respond to the noxious stimuli [3], [10], [11]. Findings were quite similar among these studies suggesting that these heroin vaccines have generally similar efficacies. Compensation in drug self-administration has not been observed with other heroin/morphine vaccines but dose-response protocols have not been performed. Compensation in nicotine and cocaine self-administration has been seen after vaccination with nicotine or cocaine conjugate vaccines [29], [30], similar to what is observed with HSA after naltrexone injection in the brain [31].

A limitation of the pharmacokinetic experiment is that heroin was administered as evenly spaced doses, in contrast to the less regular dosing pattern during self-administration. We chose this design rather than using yoked controls to minimize variability in the measured levels. Because heroin is metabolized rapidly to 6-AM and morphine, individual differences in heroin dosing patterns just prior to blood sampling for drug level measurement would lead to differences in drug levels that might not be representative of the entire 2-hr self-administration session.

Another limitation of this study is that heroin is metabolized to 6-AM more rapidly in rats than in humans, although it is rapid in both [10], [28], [32]. Because antibodies generated by M-KLH readily bind both heroin and 6-AM in plasma it is unclear if this difference would be of consequence in humans. In addition, vaccination may block heroin-induced reinstatement but would not be expected to alter cue- or stress-induced reinstatement [11]. Nevertheless, results from the current study as well as others suggest that vaccination against heroin can attenuate a number of key addiction-related behaviors in rodents. High antibody levels and antibody to drug ratios may, however, be needed for these vaccines to be most effective.

## Supporting Information

S1 Table **Opioid concentrations (mean ± SD) in plasma 4 min after the 1^st^ infusion and plasma and brain 4 min after the 8^th^ infusion of heroin in KLH (n = 5–6) and M-KLH (n = 6) rats.** *p<0.05, **p<0.01, and ***p<0.001 compared to KLH controls. Data corresponds to values shown in Fig. 4.
(DOCX)
