# Correction: Pharmacokinetic Correlates of the Effects of a Heroin Vaccine on Heroin Self-Administration in Rats

**DOI:** 10.1371/journal.pone.0126889

**Published:** 2015-04-20

**Authors:** The PLOS ONE Staff

The abbreviation for Herion self-administration (HSA) is incorrectly written as HAS in two locations in the Materials and Methods section. The subheading for section 2.9.1 should read: Acquisition of HSA. The subheading for section 2.9.2 should read: Dose-reduction and reinstatement of HSA. The publisher apologizes for this error.
