# Supplementary material for: Pharmacokinetic Correlates of the Effects of a Heroin Vaccine on Heroin Self-Administration in Rats
Source: PLoS One. 2014 Dec 23;9(12):e115696. doi: 10.1371/journal.pone.0115696 (PMC4275252; doi:10.1371/journal.pone.0115696)
Supplement: S1 Table — Opioid concentrations (mean ± SD) in plasma 4 min after the 1st infusion and plasma and brain 4 min after the 8th infusion of heroin in KLH (n = 5–6) and M-KLH (n = 6) rats. *p<0.05, **p<0.01, and ***p<0.001 compared to KLH controls. Data corresponds to values shown in Fig. 4. (DOCX) [file pone.0115696.s001.docx]

**Supporting information**

| **Plasma** |  | **Opioid concentrations (ng/ml)** | | |
| --- | --- | --- | --- | --- |
| 1^st^ heroin Infusion | | Heroin | 6-AM | Morphine |
| 0.125 mg/kg | KLH | 3 ± 1 | 25 ± 5 | 9 ± 2 |
|  | M-KLH | 1230 ± 651*** | 224 ± 144** | 148 ± 109* |
| 0.0625 mg/kg | KLH | 4 ± 1 | 14 ± 4 | 5 ± 2 |
|  | M-KLH | 1340 ± 349*** | 90 ± 80* | 25 ± 24 |
| 8^th^ heroin infusion | |  |  |  |
| 0.125 mg/kg | KLH | 7 ± 8 | 144 ± 134 | 175 ± 121 |
|  | M-KLH | 273 ± 156** | 1350 ± 1090* | 1080 ± 501** |
| 0.0625 mg/kg | KLH | 3 ± 2 | 48 ± 8 | 39 ± 14 |
|  | M-KLH | 278 ± 160** | 1360 ± 646*** | 752 ± 203*** |
| **Brain** |  | **Opioid concentrations (ng/g)** | | |
| 8^th^ heroin infusion | | Heroin | 6-AM | Morphine |
| 0.125 mg/kg | KLH | 32 ± 58 | 571 ± 301 | 106 ± 27 |
|  | M-KLH | 8 ± 7 | 423 ± 118 | 71 ± 16* |
| 0.0625 mg/kg | KLH | 3 ± 3 | 364 ± 66 | 71 ± 6 |
|  | M-KLH | 3 ± 2 | 190 ± 76** | 27 ± 14*** |

**Table S1:** Opioid concentrations (mean ± SD) in plasma 4 min after the 1^st^ infusion and plasma and brain 4 min after the 8^th^ infusion of heroin in KLH (n=5-6) and M-KLH (n=6) rats. *p < 0.05, **p < 0.01, and ***p < 0.001 compared to KLH controls. Data corresponds to values shown in Figure 4.
